# Long-term outcomes in two adult siblings with Fucosidosis – Diagnostic odyssey and clinical manifestations

**DOI:** 10.1016/j.ymgmr.2023.101009

**Published:** 2023-09-27

**Authors:** Nuria Puente-Ruiz, Ian Ellis, Marsel Bregu, Cliff Chen, Heather J. Church, Karen L. Tylee, Shalini Gladston, Richard Hackett, Andrew Oldham, Surinder Virk, Christian Hendriksz, Andrew A.M. Morris, Simon A. Jones, Karolina M. Stepien

**Affiliations:** a Adult Inherited Metabolic Diseases, Salford Royal NHS Foundation Trust, UK; b Department of Clinical Medicine, Marqués de Valdecilla University Hospital, López-Albo Post Residency Program, Santander, Spain; c Clinical Genetics, Royal Liverpool Children Hospital, Alder Hey, Liverpool, UK; d Ophthalmology Department, Warrington Hospital, Warrington, UK; e Clinical Neuropsychology Department, Salford Royal Hospital NHS Foundation Trust, Salford, UK; f Willink Metabolic Unit, St Mary's Hospital, Manchester Foundation Trust, Manchester, UK; g Warrington Primary Care Psychiatry, Warrington, UK; h Neurology Department, Salford Royal Hospital NHS Foundation Trust, Salford, UK; i Cardiology Department, Warrington Hospital, Warrington, UK; j University of Pretoria, Steve Biko Academic Unit, Department of Paediatrics, Pretoria, South Africa; k Division of Cardiovascular Sciences, University of Manchester, Manchester M13 9PL, UK

**Keywords:** Fucosidosis, Angiokeratomas, Learning disability, Long-term outcomes, Natural history

## Abstract

Fucosidosis (OMIN# 230000) is a rare lysosomal storage disorder (LSDs) caused by mutations in the *FUCA1* gene, leading to alpha-L-fucosidase deficiency; it is inherited as an autosomal recessive trait. Fucosidosis represents a disease spectrum with a wide variety of clinical features, but most affected patients have slow neurologic deterioration. Many patients die young and the long-term clinical outcomes in adult patients are poorly documented. Here, we report the long-term follow up of two Caucasian siblings, a 31-year-old man and 25-year-old woman.

We describe the clinical, biochemical, radiological and genetic findings in two siblings affected by Fucosidosis and the differences between them after 19-years follow up. The dermatological features of the younger sibling have been reported previously by Bharati et al. (2007).

Both patients have typical features of Fucosidosis, such as learning difficulties, ataxia, and angiokeratomas with differing severity. Case 1 presents severe ataxia with greater limitation of mobility, multiple dysostoses, angiokeratomas on his limbs, retinal vein enlargement and increased tortuosity in the eye and gastrointestinal symptoms. Biochemical analysis demonstrated a deficiency of alpha-fucosidase in leucocytes. Case 2 has a greater number of angiokeratomas and has suffered three psychotic episodes. The diagnosis of Fucosidosis was confirmed in cultured skin fibroblast at the age of 12 years. Molecular analysis of the *FUCA1* gene showed a heterozygous mutation c.998G > A p.(Gly333Asp), with a pathogenic exon 4 deletion in the other allele in both patients.

Conclusion. Fucosidosis presents a wide clinical heterogeneity and intrafamilial variability of symptoms. Psychosis and gastrointestinal symptoms have not been reported previously in Fucosidosis.

## 1 Introduction

Fucosidosis (OMIM# 230000) is an autosomal recessive lysosomal storage disease (LSD) caused by biallelic germline mutations of the *FUCA1* gene. This is localized on chromosome 1p34.11-1p36.11 and contains eight exons and seven introns [[1], [2], [3], [4]]. The *FUCA1* gene encodes a 461 aminoacid homotetramer protein called α-L-fucosidase [1,2,5]. Homozygous or compound heterozygous mutations of *FUCA1* gene lead to α-L-fucosidase (EC 3.2.1.51) deficiency [6].

Fucose (C6H12O6) is a deoxy sugar found in most plasma glycoproteins and in tissue mucopolysaccharides and mucolipids [1,7]. Deficiency of α-L-fucosidase causes incomplete catabolism of N- and O-glycosylproteins. As a result, fucose-containing compounds accumulate in lysosomes in tissues throughout the body [1,8,9].

Fucosidosis has an incidence below 1 in 200,000 live births [10]. To date, Fucosidosis has been identified in more than 20 countries with the highest incidence in southern Italy, the southwestern United States, the Hispanic-American population of New Mexico and Colorado, and Cuba [1,[11], [12], [13], [14], [15]].

Fucosidosis has previously been divided into types 1 and 2 according to the age of onset and clinical symptoms but nowadays the disease is considered to have a spectrum of severity and a wide variety of expressions. The mean age of the first symptom presentation is 1.2 ± 0.8 years [2]. Early onset cases (before the age of one year) with rapid progression were labeled Fucosidosis type I, while those labeled Fucosidosis type II had milder symptoms, slower progression, and longer survival [1,16,17].

Infants with severe clinical features have a neurodegenerative disorder with progressive motor impairment, including delayed development of skills required to coordinate mental and muscular exercises. They have also a progressive neurological deterioration with intellectual disability and uncontrolled rigid extensions and rotations of the limbs [18]. Seizures may be present in up to 38% of all cases [2].

Coarse faces, thickened lips, enlarged tongue, respiratory tract infections (in up to 78%), mild dysostosis multiplex (58%), mild mitral regurgitation (50%), and growth retardation are common physical findings [2]. Eye abnormalities could be found due to storage material in conjunctival, retinal, and skin vessels: dilated and tortuous retinal veins (54%), dilated and tortuous conjunctival vessels (41%), corneal opacities (11%), and pigmentary retinopathy (7%) [1,2,17].

Individuals with less severe forms and longer survival may develop telangiectasia on the skin or conjunctiva and widespread angiokeratomas, mainly on the abdomen, buttocks, thighs, and external genitalia. Angiokeratomas are present in up to 50% of affected individuals, they often progress with age and their presence increases the probability of diagnosis [1,2,19].

Less common findings include hepatomegaly (20–40%), splenomegaly (25%), cardiomegaly, or elevated sweat sodium chloride (especially in severe forms) [1,20,21].

More than 130 cases of Fucosidosis have been reported worldwide [22]. Most of the affected individuals described in the literature are children and the long-term clinical outcomes in alive adult patients are unknown. Here, we report the long-term follow up of two Caucasian siblings. The dermatological features of the younger sibling have been reported previously [23].

## 2 Methods

We describe the clinical, biochemical, radiological and genetic findings in two siblings affected by Fucosidosis, who were followed-up in metabolic clinics for 19-year.

### 2.1 Case 1

This boy was born at 36 weeks gestation (birth weight 3.005 Kg) and had normal developmental milestones. In childhood he required an umbilical hernia repair. He presented in infancy with red skin lesions, gradually increasing in number.

In primary school he was noted to have academic problems from an early age and an educational assessment showed him to be approximately a year behind his peer group. He demonstrated specific interests in sports statistics (being a football club fan) and knew bus routes by heart. Although he had a limited friendship circle, he enjoyed socializing. At the age of 12, he was referred to a Clinical Geneticist to investigate his learning difficulties and a red mark on the back of his ear [23].

Referral for a lysosomal enzyme screen identified deficient alpha-L-fucosidase activity in mixed leucocytes, suggesting a biochemical diagnosis of Fucosidosis. This was subsequently confirmed by enzyme analysis in cultured skin fibroblasts that also showed a marked deficiency of alpha-L-fucosidose (2.2 nmol/mg/h; in assay normal controls 17 & 54 nmol/mg/h) concordant with previous results and consistent with a biochemical diagnosis of Fucosidosis type II.

Molecular analysis of the *FUCA1* gene (LSD gene panel) showed a heterozygous mutation c.998G > A p.(Gly333Asp) in the *FUCA1* gene. A few years later, when whole genome sequencing became available, a repeat analysis additionally identified a pathogenic exon 4 deletion in the *FUCA1* gene.

At the age of 20 years, he developed low back pain. He has had a few respiratory infections as an adult. He reported occasional gastrointestinal dysfunction and diarrhoea shortly after meals. He struggled to organise his work, finances, and to keep track of day-to-day memories. However, he could maintain paid employment by following simple instructions and performing tasks with oversight from his colleagues. The job provided him with a sense of wellbeing and purpose, as well as a social outlet several times per week. He was not reported to have suffered with anxiety, depression or behavioural difficulties.

At 31 years, on clinical examination his blood pressure was 100/70 mmHg. He has a grade 2/4 ejection murmur in the aortic valve. His jugular venous pressure was not raised and his chest was clear to auscultation. He did not have any abnormal respiratory findings and the infection rate was not increased. He has a broad-based gait and ataxia. Scale for the Assessment and Rating of Ataxia (SARA) score was used to determine the severity of ataxia; scored 16/40, with a tendency to scissor with his legs. Orthotic devices have improved the externally rotated position of his feet. He can climb stairs using 2 handrails.

Although the initial echocardiogram showed mixed aortic valve disease with some thickening of his aortic valve, mild aortic stenosis (maximum velocity 2.6 m/s, mean pressure gradient 14 mm/Hg) and mild aortic regurgitation, he remained asymptomatic and subsequent echocardiograms did not show any progression of his valvular disease. There was no left ventricular hypertrophy.

**Fig. 1:**
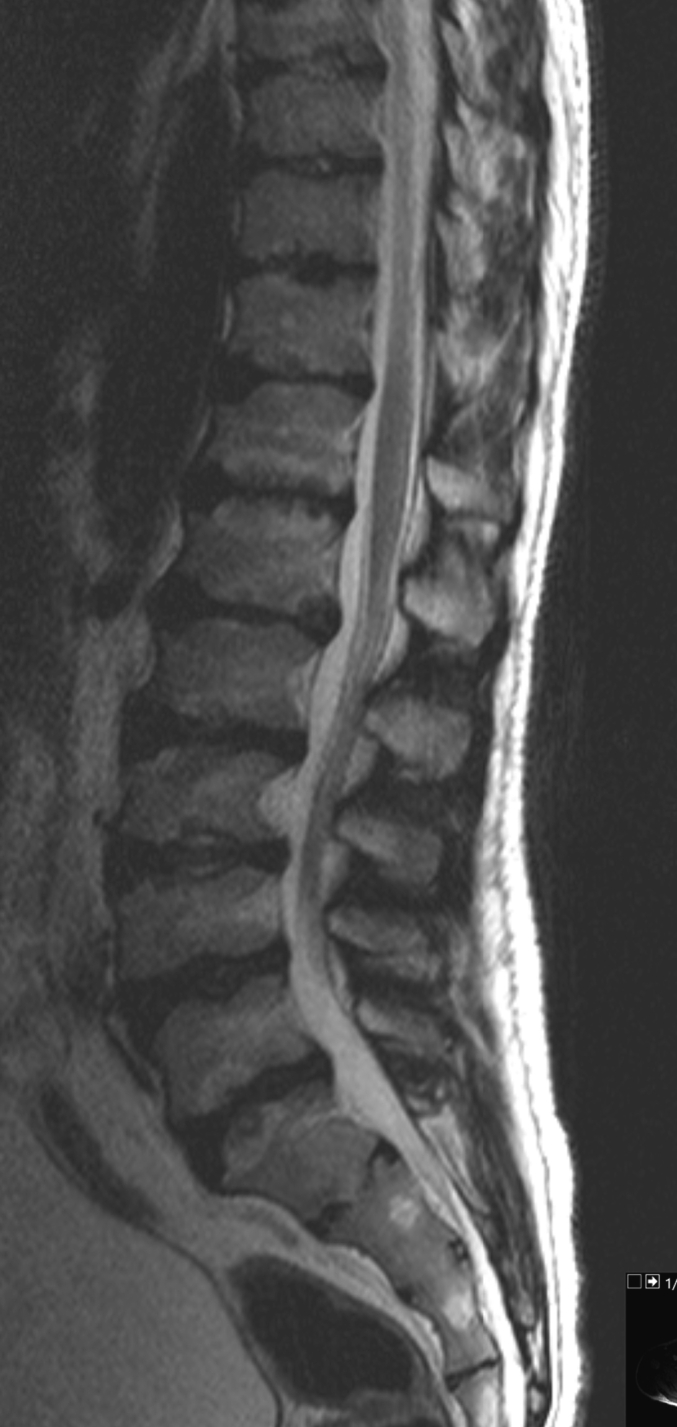
a - MRI spine: dysostosis multiplex in Case 1 (age 20); b- Dysostosis multiplex in lumbar spine in Case 1 (age 20).

The changes in his skeletal system were consistent with dysostosis multiplex (Fig. 1). MRI of his spine showed a normal cranio-cervical junction, but multiple abnormalities within the vertebral column.

He had fewer angiokeratoma corporis diffusum as compared to his sister.

**Fig. 2:**
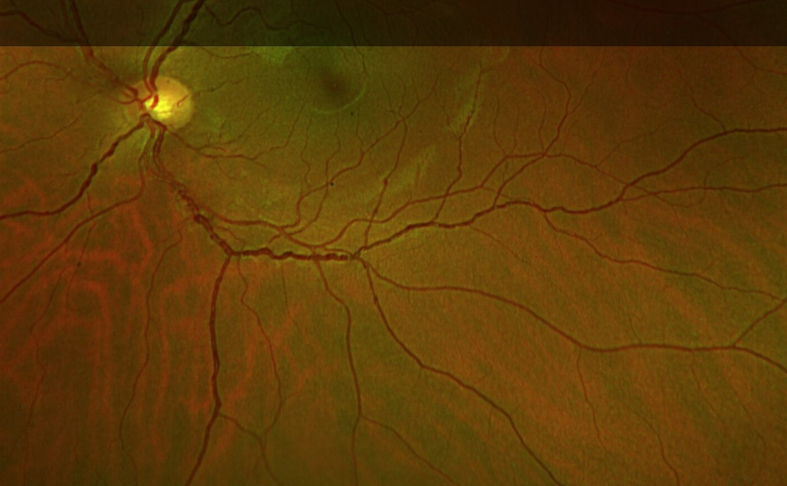
Changes in retinal veins in Case 1 (age 31).

His ophthalmic assessment showed mild retinal vein enlargement and increased tortuosity, but his vision has remained stable (Fig. 2).

He had no organomegaly, and his abdominal ultrasound showed the liver has smooth contour and homogenous echotexture. His synthetic liver function was evaluated at different time points and was consistently normal.

His latest neurocognitive assessment showed all scores in the impaired range (<5th percentile), apart from a low average score on one verbal comprehension task involving following simple commands (9th percentile). At interview he had significant speech difficulties, but occasionally uttered full sentences. His memory was variable. He needed prompts to recall information, sometimes failed to recognize work colleagues when he met them around the town and did not recall the plotlines of TV shows. His parents supported him with managing his finances and his night-shifts at the local post office. Although he was independent when using buses around the local town centre, this had been a special interest of his for many years. Data were not available on his previous intellectual assessments, but the family reported no evidence of regression.

### 2.2 Case 2

This girl was born at term (birth weight 2.95 Kg) and had a small ventricular septal defect, which closed after a septal defect cardiac surgery. She had delayed speech, for which she required speech therapy.

**Fig. 3:**
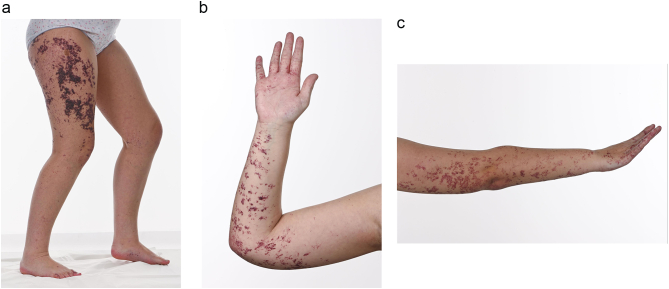
a/b/c- Angiokeratoma corporis diffusum on the right thigh, right palm, and right forearm (Case 2).

She was confirmed to be affected with Fucosidosis at the age of 6 years, after the diagnosis in her brother. She had learning difficulties in childhood and she had diffuse confluent angiokeratomas on the skin of her limbs, trunk and buttock (Fig. 3). They had a migratory pattern, bled on trauma, but were not itchy and imposed a therapeutic challenge to the dermatology team at the time of diagnosis which was previously documented in 2007 [23].

**Fig. 4:**
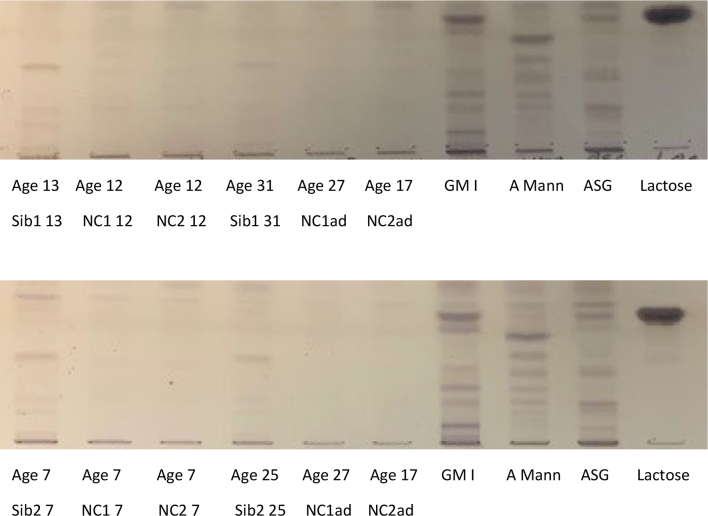
Urine oligosaccharides in two siblings at diagnosis and in adulthood.

She started mainstream primary school, started to read and write but there was some deterioration in her skills. She has received extra support at school since the age of 10 years.

There have been no seizures, but she has mild hearing loss in one ear. She has had no skeletal problems.

At the age of 14 years, there was a decline in her cognitive function following an infection with a subsequent partial recovery. During this episode, she had weight loss, changes in her sleep pattern, hallucinations and agitation. She talked to herself and had repeated vocalizations of a semi-purposeful nature. All the symptoms improved after few months without any specific treatment. No organic cause was found; basic biochemical investigations and an EEG were normal. It was concluded that she has suffered an episode of psychosis as part of her underlying Fucosidosis. Following the weight loss during the acute illness, her menstruation ceased and came back after her body weight improved.

She had another episode of psychosis aged 16 years, with full recovery. She dresses herself but she needs supervision of the water temperature in the shower. She can take herself to the toilet but there have been occasional problems with soiling and wetting. She has no problems with aggression or irritability.

Around the time of the transition to adult metabolic services, she was in mainstream school with one-to-one support. Prior to the episodes of psychosis, she was able to use gadgets such as iPads and television, perform kitchen tasks under supervision, spell and write simple phrases, and perform dance routines. She could recount episodes from her day at college using simple language.

A third episode of psychosis occurred just after her 21st birthday. Her parents reported aggression but no self-harm, disinhibition, auditory or visual hallucinations. After starting therapy with antipsychotics, Diazepam for agitation and Zopiclone to aid sleep, all the symptoms improved. The episode lasted approximately 12 months. Diazepam and Zopiclone were discontinued (age 22), and she continued on Quetiapine, remaining stable from a mental health point of view. It was hypothesized that stressful events or changes in her routines might have played a part in precipitating these disturbances.

Over a few months after her third episode, her cognitive and behavioural problems gradually improved, albeit with some episodes of tearfulness and aggression from which she quickly recovered. Her presentation improved after treatment with Quetiapine 50 mg daily, returning to her baseline. She attended college, began performing dance routines, and her writing and spelling improved to the point where she was using subject-verb phrases.

Neurocognitive assessment at 22 years of age showed that her verbal comprehension index (<1st percentile), perceptual reasoning index (<1st percentile) and her full-scale IQ (<1st percentile) were all in the impaired range (Supplementary Table 2). On language tasks she demonstrated good knowledge of words (e.g. ‘bird’ and ‘finger’), and even simple abstract conceptualizations. For instance, she identified superordinate categories for concrete words e.g. ‘cow’ and ‘pig’ belonged in the category ‘animal’; but she failed to identify more abstract super-ordinate categories, e.g. ‘ice cream’ and ‘chocolate’ belonged to the category ‘sweet’. The accuracy of her visual search and sequencing improved, and her motor speed increased from impaired to low average. She could solve problems by matching the orientation of shapes to their target designs, but was unable to solve problems using more than one attribute e.g. orientation and colour. She engaged in more eye-contact with the examiner and required less prompting to carry out tasks. Data on any previous intellectual assessments were not available for comparison, but at the feedback appointment, her parents felt that the above neurocognitive assessment was a good reflection of her prior baseline.

The physiotherapy assessment showed that she had also mild ataxia with a SARA scale score of 6/40, and she had only higher level balance difficulties. There were no issues with mobility, she was able to walk without support and to climb stairs. She enjoys various activities, including dancing.

There were no swallowing problems or gastrointestinal dysfunction to report. Her abdominal ultrasound was unremarkable.

Her ophthalmology assessment showed retinal vein enlargement and increased tortuosity in her retinal vessels, but no other changes.

Her endocrine function was normal (Table 1); she has had regular menstruation since the age of 12 years.

**Table 1:** Differences in clinical outcomes between both siblings affected with Fucosidosis t II.

	Case 1	Case 2
Sex, Current Age	Male, 31y	Female, 25y
Height /BMI	161.2 cm/ 2.9th Centile	151.1 cm/ 6.3th Centile
	22.9 kg/m^2^	35.6 kg/m^2^
Age at diagnosis	12 years	6 years
Enzyme (Leucocytes)	0.22 nmol/mg/h (50–250)	-
Enzyme (Fibroblasts)	2.2 nmol/mg/h (in assay normal values: 17 and 54)	1.1 nmol/mg/h (in-assay normal values 17 and 54)
Urine oligos	see Fig. 4	see Fig. 4
Urine GAGs	normal	normal
Genetic mutation	c.998G > A p.(Gly333Asp) in the *FUCA1* gene, a pathogenic exon 4 deletion in the *FUCA1* gene.	c.998G > A p.(Gly333Asp) in the *FUCA1* gene, a pathogenic exon 4 deletion in the *FUCA1* gene.
Physio assessment:		
SARA ataxia scale	16/40	6/40
Hip strength	5/5 (bi-laterally)	5/5
Spinal position	Slightly hyperlordotic	No abnormalities
Neurocognitive assessment⁎	Impaired ranges (<5th percentile), except receptive language (9th percentile)	Impaired ranges (<1st percentile), except motor speed (9th percentile)
Visual acuity: Intraocular pressure:	Right 0.60 (6/24) with glasses. Left 0.80 (6/38) with glasses Right eye IOP 10 mmHg and left eye IOP 11 mmHg Fundus: Bilateral extensive coiling of retinal vessels with venous dilatation	Right 0.14 (6/7.5) unaided; Left 0.16 (6/9.5) unaidedFundus: Bilateral extensive coiling of retinal vessels with venous dilatation
Cardiac:		
ECG:	SR 68/min	SR 72/min
Echocardiogram:	no cardiomyopathy	no cardiomyopathy
	EF 80%	EF 70%
Pulmonary function tests:	FEV1 1.94 L (52% predicted)	FEV1 2.13 L (37.7%)
	FVC% 2.47 L (FVC 57% predicted)	FVC 2.46 L (43.8% predicted)
Gastrointestinal:	Occasional diarrhoea	None
Abdominal US:	No hepatosplenomegaly	No hepatosplenomegaly
Skeletal abnormalities:		
X ray spine:	Kyphoscoliosis	N/A
MRI:	Dysostosis multiplex	N/A
Endocrine:		
Thyroid function test:	normal	normal
Gonadotrophins:	normal	normal

⁎ Wechsler Intelligence Scales (Wechsler Abbreviated Scale of Intelligence-II & Wechsler Adult Intelligence Scale-IV); Delis-Kaplan Executive Function System; Multilingual Aphasia Examination; Visual Object and Space Perception battery; Hayling & Brixton; Repeatable Battery for the Assessment of Neuropsychological Status; N/A not available.

## 3 Discussion

There are few case reports of the long-term outcomes in adults with Fucosidosis and it widely thought that few patients survive beyond 30 years. Willems et al. showed that only 64% of Fucosidosis patients reached the second decade in a cohort of 77 patients [2]. Another case series describes 10 patients from 6 unrelated families. Patients were diagnosed at a mean age of 29 ± 10.3 months. They had severely delayed early motor development and 6 of the 10 patients died before 10 years of age [24].

Clinical variability has been reported in the literature, but most affected patients have slow neurologic deterioration [2]. Fleming et al described a 46-year-old woman, who had severe muscle wasting, several minor long-bone fractures, was unable to walk, and had lost all verbal and most nonverbal communication. She required total care support, but there had been little evident progression since the diagnosis at the age of 20 years, when she suffered from physical and mental retardation, short stature, angiokeratoma corporis diffusum, dysostosis multiplex and generalised muscle wasting [25].

The clinical heterogeneity observed in these families might not only be the result of variants in *FUCA1* gene. Other unknown modifying genes or environmental factors may contribute to expression of different clinical features [26]. This is supported by the intrafamilial variability reported in patients with Fucosidosis in two families [27]. Affected individuals had a wide range of symptoms and different rates of progression.

Despite carrying the same mutations and sharing some clinical features, the long-term complications differ in our two patients. Both patients show typical features of Fucosidosis, such as learning difficulties, ataxia, and angiokeratomas. Case 1, however, has more severe ataxia with greater limitation of mobility, multiple dysostosis, retinal vein enlargement and increased retinal vein enlargement and increased vein tortuosity, and gastrointestinal symptoms; the latter have not previously been reported in Fucosidosis but they are seen in other LSDs such as Fabry disease, Pompe disease, Mucopolysaccharidosis and Niemann Pick type C. Case 2 has more wide spread angiokeratomas and she has suffered three psychotic episodes.

Oligosaccharide disorders share many features in common with other LSDs. The origin of gastrointestinal symptoms in LSDs is complex and triggered by many factors. Diarrhoea is the second most common gastrointestinal symptom in LSDs and is seldom associated with blood or mucus in the stool [28]. The diarrhoea may be due to neuropathy, myopathy, an unbalanced entero-hepatic circulation of bile acids or substrate deposits in the intestinal mucosa. These factors may cause hyperactive uncoordinated contractions or gut inflammation [29].

Psychotic symptoms have not previously described in Fucosidosis, but a number of LSDs present with neuropsychiatric symptoms at various stages of the disease process such as Alpha-mannosidosis, Niemann-Pick type C or later onset forms of GM2 gangliosidosis [30,31]. Oligosaccharidoses may have psychotic symptoms in adult or adolescent-onset forms, and they are indistinguishable from typical psychotic presentations of schizophrenia [31]. Psychiatric symptoms may even be the first manifestation of oligosaccharidoses [[32], [33], [34], [35]].

Malm et al described detailed psychotic episodes and behavioural problems in 57 out of 125 patients with Alpha-mannosidosis. The psychotic disorders had an onset in late puberty or early adolescence and they became more frequent with age. Behavioural and psychotic issues were described in 20% of patients aged under 10, and in 70% of patients older than 30. The psychotic episodes were often triggered by psychological stress and followed by cognitive loss [36].

Oligosaccharidoses are generally associated with skeletal abnormalities, except for patients with Sialidosis type I who have normal skeletal anatomy [37]. In addition to osteoporosis, thin bone cortices and delayed skeletal maturation, most patients with Fucosidosis develop mild dysostosis multiplex involving the axial and appendicular skeleton. Dysostosis multiplex has been described in 58% of patients and kyphoscoliosis in 66% of Fucosidosis patients [2]. Radiological studies often reveal a kyphosis caused by deformity of the anterosuperior portion of the vertebral bodies, [38] and dysostosis multiplex signs, involving spine, pelvis, the short tubular bones of the hands and thickened calvaria [39]. Kyphosis or scoliosis has been reported in 33% of Alpha mannosidosis patients aged up to 30 years and radiological findings show mild-to-moderate dysostosis multiplex in 90% of patients [36]. Sialidosis type II is also another oligosaccharidoses characterised by early-onset skeletal abnormalities [37].

Dilated and tortuous retinal veins (54%) and conjuctival vessels (53%) are the most common described ocular abnormalities [2]. Only subtle ophthalmic changes were observed in our patients. Tortuosity of the ophthalmic veins are not typical or confined to Fucosidosis.

Cardiomyopathy is not a typical feature in Fucosidosis patients and not observed in our two siblings, although cardiac involvement was observed in other oligosaccharidoses [40].

Currently, preclinical studies are underway to test the effectiveness of Intracisternal Enzyme Replacement Therapy (ERT) for Fucosidosis. Administering the enzyme directly to the central nervous system (CNS) has been shown to partially improve neuropathology [1,41]. Hematopoietic-stem-cell transplantation (HSCT) showed α-L-fucosidase enzyme activity normalized in leucocytes, plasma, and neural and visceral tissues in dogs [1,48,49]. HSCT has been performed in a few selected patients [42]. As one might expect, the treatment is most effective in presymptomatic patients. Improvements in MRI findings, psychomotor development, swallowing and the number of respiratory tract infections have been reported but not in the dysostosis multiplex [43].

Thanks to advances in diagnostic technology and sequencing techniques, the number of patients diagnosed with Fucosidosis has increased. So far 35 pathogenic variants in the *FUCA1* gene have been described in HGMD [44] along with 42 pathogenic variants (some overlap of described mutations will be apparent between HGMD and ClinVar), 12 likely pathogenic, 140 VUS and 10 controversial have been described in ClinVar [45]. Most of them (11) are nonsense mutations and 6 are missense. Only three splice site variants have been reported [1,44,46,47]. Nine small deletions, one small insertion, one gross insertion and one stop-loss mutation have also been reported [1,48]. Two novel homozygous deletions lead to a frameshift, resulting in the formation of a truncated *FUCA1* protein [14,15].

The two variants found in *FUCA1* are likely to be the cause of the Fucosidosis in these siblings. The exon 4 deletion was undoubtedly pathogenic; it causes a frameshift and has previously been reported [45]. The c.998G > A p.(Gly333Asp) has been reported previously as a variant of uncertain significance [45]. Algorithms developed to predict the effect of missense changes on protein structure and function do not agree on the potential impact of this missense change (SIFT: “Deleterious”; PolyPhen-2: “Probably Damaging”; Align-GVGD: “Class C15”) [49].

## 4 Conclusions

Fucosidosis shows considerable clinical variation between individuals with different mutations and also within families, although these subjects share the same mutations. Personalized care with the aim of preventing complications has increased the survival of affected patients. The pathophysiology of the psychotic episodes in our patient with Fucosidosis is uncertain but similar episodes have been reported in others lysosomal storage disorders.

## Contributorship

All the authors contributed to the study design, data collection and the manuscript writing. They all approved the final version of the manuscript.

Guarantor: KMS.

## Ethical statements

The study was conducted according to the guidelines of the Declaration of Helsinki.

The patient's parents provided written consent to participate in genetic test.

Consent for publication: Written consent to publish this information has been obtained from the patients and parents.

## Funding

None.

## Declaration of Competing Interest

all the authors declare no conflict of interest for this publication.

## Data Availability

The data that has been used is confidential.

## Appendix A Supplementary data

Supplementary Table 2 Neuropsychology assessment; types of tests and their scores. Supplementary Table 2

## References

[1] Stepien K.M., Ciara E., Stanek A.J. (2021). Fucosidosis-clinical manifestation, long term outcomes, and genetic profile review and case series. Genes (Basel).

[2] Willems P.J., Gatti R., Darby J.K. (1991). Fucosidosis revisited: a review of 77 patients. Am. J. Med. Genet..

[3] Kretz K.A., Cripe D., Carson G.S., Fukushima H., O’Brien J.S. (1992). Structure and sequence of the human α-L-fucosidase gene and pseudogene. Genomics.

[4] Fukushima H., de Wet J.R., O’Brien J.S. (1985). Molecular cloning of a cDNA for human alpha-L-fucosidase. Proc. Natl. Acad. Sci. U. S. A..

[5] Ip P., Goh W., Chan K.W. (2002). A novel FUCA1 mutation causing fucosidosis in a Chinese boy. J. Inherit. Metab. Dis..

[6] Van Hoof F., Hers J.G. (1968). Mucopolysaccharidosis by absence of α-fucosidase. Lancet.

[7] Durand P., Borrone C., Della Cella G. (1969). Fucosidosis. J. Pediatr..

[8] Johnson S.W., Piesecki S., Wang R.F., Damjanov I., Alhadeff J.A. (1992). Analysis of purified human liver α-L-fucosidase by western-blotting with lectins and polyclonal and monoclonal antibodies. Biochem. J..

[9] Michalski J.C., Klein A. (1999). Glycoprotein lysosomal storage disorders: α- and β-mannosidosis, fucosidosis and α-N-acetylgalactosaminidase deficiency. Biochim. Biophys. Acta.

[10] Gowda V.K., Srinivasan V.M., Vegda H., Bhat M. (2020). Fucosidosis with pathogenic variant in FUCA1 gene. Indian J. Pediatr..

[11] Willems P.J., Seo H.C., Coucke P., Tonlorenzi R., O’Brien J.S. (1999). Spectrum of mutations in fucosidosis. Eur. J. Hum. Genet..

[12] Chkioua L., Amri Y., Chaima S., Fenni F., Boudabous H., Ben Turkia H., Messaoud T., Tebib N., Laradi S. (2021). Fucosidosis in Tunisian patients: mutational analysis and homology-based modeling of FUCA1 enzyme. BMC Med. Genet..

[13] Panmontha W., Amarinthnukrowh P., Damrongphol P. (2016). Novel mutations in the FUCA1 gene that cause fucosidosis. Genet. Mol. Res..

[14] Domin A., Zabek T., Kwiatkowska A., Szmatola T., Deregowska A., Lewinska A., Mazur A., Wnuk M. (2021). The identification of a novel fucosidosis-associated FUCA1 mutation: a case of a 5-year-old polish girl with two additional rare chromosomal aberrations and affected DNA methylation patterns. Genes (Basel).

[15] Zhang X., Zhao S., Liu H., Wang X., Wang X., Du N., Liu H., Duan H. (2021). Identification of a novel homozygous loss-of-function mutation in FUCA1 gene causing severe fucosidosis: a case report. J. Int. Med. Res..

[16] Saudubray J.M., Baumgartner M., García-Cazorla Á., Walter J. (2016). Diagnosis and Treatment.

[17] Tiberio G., Filocamo M., Gatti R., Durand P. (1995). Mutations in fucosidosis gene: a review. Acta Genet. Med. Gemellol..

[18] Grabowski G.A. (2003). NORD Guide to Rare Disorders.

[19] Kulcsarova K., Baloghova J., Necpal J., Skorvanek M. (2022). Skin conditions and movement disorders: hiding in plain sight. Mov. Disord. Clin. Pract..

[20] Mao S.J., Zhao J., Shen Z., Zou C.C. (2022). An unusual presentation of fucosidosis in a Chinese boy: a case report and literature review (childhood fucosidosis). BMC Pediatr..

[21] Kaur A., Dhaliwal A.S., Raynes H., Naidich T.P., Kaufman D.M. (2019). Diagnosis and supportive management of fucosidosis: a case report. Cureus.

[22] Do Rosario M.C., Purushothama G., Narayanan D.L., Siddiqui S., Girisha K.M., Shukla A. (2023). Extended analysis of exome sequencing data reveals a novel homozygous deletion of exons 3 and 4 in FUCA1 gene causing Fucosidosis in an Indian family. Clin. Dysmorphol..

[23] Bharati A., Higgins C., Ellis I., Wraith J. (2007). Fucosidosis: a therapeutic challenge. Pediatr. Dermatol..

[24] Turkia H.B., Tebib N., Azzouz H., Abdelmoula M.S., Bouguila J., Sanhaji H. (2008). Phenotypic spectrum of fucosidosis in Tunisia. J. Inherit. Metab. Dis..

[25] Fleming C.J., Sinclair D.U., White E.J., Winchester B., Whiteford M.L., Connor J.M. (1998). A fucosidosis patient with relative longevity and a missense mutation in exon 7 of the alpha-fucosidase gene. J. Inherit. Metab. Dis..

[26] Beck M. (2001). Variable clinical presentation in lysosomal storage disorders. J. Inherit. Metab. Dis..

[27] Willems P.J., Garcia C.A., De Smedt M.C.H., Martin-Jimenez R., Darby J.K., Duenas D.A., Granado-Villar D., O’Brien J.S. (1988). Intrafamilial variability in fucosidosis. Clin. Genet..

[28] Göktaş M.A., Gümüş E., Demir H., Gülşen H.H., Saltık-Temizel I.N., Özen H., Güçer S., Yüce A. (2021). A very rare cause of protein losing enteropathy: Gaucher disease. Turk. J. Pediatr..

[29] Caputo F., Lungaro L., Galdi A., Zoli E., Giancola F., Caio G., De Giorgio R., Zoli G. (2021). Gastrointestinal involvement in anderson-fabry disease: a narrative review. Int. J. Environ. Res. Public Health.

[30] Rego T., Farrand S., Goh A.M.Y., Eratne D., Kelso W., Mangelsdorf S., Velakoulis D., Walterfang M. (2009). Psychiatric and cognitive symptoms associated with Niemann-pick type C disease: neurobiology and management. CNS Drugs.

[31] Ong L.T. (2021). Psychosis symptoms associated with Niemann-Pick disease type C. Psychiatr. Genet..

[32] Seidl U., Giesel F.L., Cantz M., Schmidbauer M., Schröder J., Pantel J. (2004). Unusual course of alpha mannosidosis with symptoms of paranoid-hallucinatory psychosis. Nervenarzt.

[33] Gutschalk A., Harting I., Cantz M., Springer C., Rohrschneider K., Meinck H.M. (2004). Adult alpha mannosidosis: clinical progression in the absence of demyelination. Neurology.

[34] Walterfang M., Bonnot O., Mocellin R., Velakoulis D. (2013). The neuropsychiatry of inborn errors of metabolism. J. Inherit. Metab. Dis..

[35] Saudubray J.M. (2009). Neurometabolic disorders. J. Inherit. Metab. Dis..

[36] Malm D., Riise Stensland H.M., Edvardsen Ø., Nilssen Ø. (2014). The natural course and complications of alpha-mannosidosis--a retrospective and descriptive study. J. Inherit. Metab. Dis..

[37] Lowden J.A., O’Brien J.S. (1979). Sialidosis: a review of human neuraminidase deficiency. Am. J. Hum. Genet..

[38] (2002). Diagnosis of Bone and Joint Disorders.

[39] Malatt C., Koning J.L., Naheedy J. (2015). Skeletal and brain abnormalities in Fucosidosis, a rare lysosomal storage disorder. J. Radiol. Case Rep.

[40] Prasanna P., Sriram C.S., Rodriguez S.H., Kohli U. (2021). Cardiovascular involvement in alpha-n-acetyl neuraminidase deficiency syndromes (sialidosis type I and II). Cardiol. Young.

[41] Kondagari G.S., Fletcher J.L., Cruz R., Williamson P., Hopwood J.J., Taylor R.M. (2015). The effects of intracisternal enzyme replacement versus sham treatment on central neuropathology in preclinical canine fucosidosis. Orphanet J. Rare Dis..

[42] Miano M., Lanino E., Gatti R., Morreale G., Fondelli P., Celle M.E., Stroppiano M., Crescenzi F., Dini G. (2001). Four year follow-up of a case of fucosidosis treated with unrelated donor bone marrow transplantation. Bone Marrow Transplant..

[43] Jiang M., Liu S., Jiang H., Lin Y., Shao Y., Hu H., Zhao X., Liu H., Huang Y., Liu L. (2017). Brain abnormalities in fucosidosis: transplantation or supportive therapy?. Metab. Brain Dis..

[44] HGMD Professional http://www.hgmd.cf.ac.uk/ac/index.php.

[45] ClinVar National Library of Medicine. https://www.ncbi.nlm.nih.gov/clinvar/variation/1957896/.

[46] Reuter M.S., Tawamie H., Buchert R., Gebril O.H., Froukh T., Thiel C., Uebe S., Ekici A.B., Krumbiegel M., Zweier C. (2017). Diagnostic yield and novel candidate genes by exome sequencing in 152 consanguineous families with neurodevelopmental disorders. JAMA Psychiatry.

[47] Wali G., Wali G.M., Sue C.M., Kumar K.R. (2019). A novel homozygous mutation in the FUCA1 gene highlighting fucosidosis as a cause of dystonia: case report and literature review. Neuropediatrics.

[48] Williamson M., Cragg H., Grant J., Kretz K., O’Brien J., Willems P.J., Young E., Winchester B. (1993). A 5′ splice site mutation in fucosidosis. J. Med. Genet..

[49] Varsome The Human Genomics Community. https://varsome.com/gene/hg38/FUCA1.

