# Supplementary material for: Long-term outcomes in two adult siblings with Fucosidosis – Diagnostic odyssey and clinical manifestations
Source: Mol Genet Metab Rep. 2023 Sep 27;37:101009. doi: 10.1016/j.ymgmr.2023.101009 (PMC10694746; doi:10.1016/j.ymgmr.2023.101009)
Supplement: Supplementary Table 2 — Neuropsychology assessment; types of tests and their scores. [file mmc1.docx]

Supplementary Table 2. Neuropsychology assessment in both siblings; types of tests used and their scores.

| **Case 1** | | | **Case 2** | | |
| --- | --- | --- | --- | --- | --- |
| ***General Intellectual function*** | | | | | |
| **Test** | **Raw score** | **Standard score** | **Test** | **Raw score** | **Standard score** |
| ToPF | 12 | 76 | WASI-II FSIQ |  | 45 |
|  |  |  |  |  |  |
| WAIS-IV |  |  | WASI-II |  |  |
| VCI |  | 58 | VCI |  | 45 |
| Similarities | 10 | 3ss | Similarities | 6 | 20T |
| Vocabulary | 7 | 3ss | Vocabulary | 12 | 20T |
|  |  |  |  |  |  |
| PRI |  | 60 | PRI |  | 52 |
| Block Design | 12 | 3ss | Block Design | 5 | 21T |
| Matrix Reasoning | 6 | 3ss | Matrix Reasoning | 7 | 24T |
| Visual Puzzles | 6 | 4ss |  |  |  |
|  |  |  |  |  |  |
| WMI |  | 58 |  |  |  |
| Digit Span | 11 | 1ss |  |  |  |
| Arithmetic | 6 | 4ss |  |  |  |
|  |  |  |  |  |  |
| PSI |  | 50 |  |  |  |
| Symbol search | 5 | 1ss |  |  |  |
| Coding | 20 | 1ss |  |  |  |
|  |  |  |  |  |  |
| ***Memory*** | | | | | |
| **Test** | **Raw score** | **Standard score** | **Test** | **Raw score** | **Standard score** |
| RBANS |  |  |  |  |  |
| Immediate memory index |  | 40 |  |  |  |
| List learning | 8 | 1ss |  |  |  |
| Story memory | 2 | 1ss |  |  |  |
|  |  |  |  |  |  |
| Delayed memory index |  | 40 |  |  |  |
| List recall | 0 | <2^nd^ %ile |  |  |  |
| List recognition | 13 | <2^nd^ %ile |  |  |  |
| Story recall | 1 | 1ss |  |  |  |
| Figure recall | 0 | 1ss |  |  |  |
|  |  |  |  |  |  |
| ***Attention*** | | | | | |
| **Test** | **Raw score** | **Standard score** | **Test** | **Raw score** | **Standard score** |
| RBANS |  |  |  |  |  |
| Attention index |  | 43 |  |  |  |
| Digit span | 6 | 2ss |  |  |  |
| Coding | 14 | 1ss |  |  |  |
|  |  |  |  |  |  |
| TEA |  |  |  |  |  |
| Elevator counting (distraction) | 1 | <5^th^ %ile |  |  |  |
|  |  |  |  |  |  |
| ***Language*** | | | | | |
| **Test** | **Raw score** | **Standard score** | **Test** | **Raw score** | **Standard score** |
| RBANS |  |  |  |  |  |
| Language index |  | 47 |  |  |  |
| Picture naming | 7 | <2^nd^ %ile |  |  |  |
| Semantic fluency | 9 | 1ss |  |  |  |
|  |  |  |  |  |  |
| MAE |  |  | MAE |  |  |
| Repetition | 7 | 1^st^ %ile | Repetition | 6 (adjusted) | <1^st^ %ile |
| Tokens test | 38 | 9^th^ %ile |  |  |  |
|  |  |  |  |  |  |
| ***Visuospatial function*** | | | | | |
| **Test** | **Raw score** | **Standard score** | **Test** | **Raw score** | **Standard score** |
| RBANS |  |  |  |  |  |
| Visuospatial index |  | 53 |  |  |  |
| Figure copy | 2 | 1ss |  |  |  |
| Line orientation | 6 | <2^nd^ %ile |  |  |  |
|  |  |  |  |  |  |
| VOSP |  |  |  |  |  |
| Incomplete letters | 14 | <5^th^ %ile |  |  |  |
| Cube analysis | 4 | <5^th^ %ile |  |  |  |
|  |  |  |  |  |  |
| ***Executive function*** | | | | | |
| **Test** | **Raw score** | **Standard score** | **Test** | **Raw score** | **Standard score** |
| DKEFS |  |  | DKEFS |  |  |
| Trail making test |  |  | Trail making test |  |  |
| Visual scanning | 68 | 1ss | Visual scanning | 60 | 1ss |
| Number sequencing | 100 | 1ss | Number sequencing | 120 | 1ss |
| Letter sequencing | 150 | 1ss | Letter sequencing | dc | dc |
| Number-letter switching | dc | dc | Number-letter switching | dc | dc |
| Motor speed | 132 | 1ss | Motor speed | 54 | 6ss |
|  |  |  |  |  |  |
| Hayling sensible completion | 49 | 5^th^ %ile |  |  |  |
| Brixton | 29 | 1^st^ %ile |  |  |  |
|  |  |  |  |  |  |

dc = discontinued as unable to perform task; DKEFS = Delis-Kaplan Executive Function System; FSQI = Full Scale Intelligence Quotient; MAE = Multilingual Aphasia Examination; PRI = Perceptual Reasoning Index; PSI = Processing Speed Index; RBANS = Repeatable Battery for the Assessment of Neuropsychological Status; ss = scaled score; T = T-score; TEA = Test of Everyday Attention; ToPF = Test of Premorbid Function; VCI = Verbal Comprehension Index; VOSP = Visual Object and Space Perception Battery; WAIS-IV = Wechsler Adult Intelligence Scale 4^th^ edition; WASI-II = Wechsler Abbreviated Scale of Intelligence 2^nd^ edition; WMI = Working Memory index
